# Capacity needs of Pakistani psychiatrists to deliver community-sensitive mental health services to sexual minority men

**DOI:** 10.1371/journal.pgph.0004948

**Published:** 2026-01-28

**Authors:** Usman Ali, Kamran Maqsood, Asad Ullah, Bariah Rafiq, Noman Tariq, Azhar Ali, Ali Madeeh Hashmi

**Affiliations:** 1 Academic Department of Psychiatry and Behavioral Science, King Edward Medical University, Lahore, Pakistan; 2 Academic Department of Psychiatry and Behavioral Science, Sheikh Khalifa bin Zayed Al Nahyan Hospital, Azad Jammu and Kashmir, Pakistan; London School of Hygiene and Tropical Medicine Faculty of Epidemiology and Population Health, UNITED KINGDOM OF GREAT BRITAIN AND NORTHERN IRELAND

## Abstract

Sexual minorities face a high degree of social and religious stigma in Pakistan. The current study aims to identify the capacity needs of the psychiatry fraternity for providing person-centered care to sexual minority men in Pakistan. A qualitative exploratory study was conducted from May 2024 to October 2024. Four consultant psychiatrists (CP), nine postgraduate residents (PGR) in psychiatry, and eight sexual minority men (SM) were interviewed. Thematic analysis was done. Data was analyzed using NVIVO version 14.0. Main emergent themes included a lack of understanding among psychiatrists related to the human rights of SM, and confidentiality, a lack of knowledge about sexuality and gender, stigma in psychiatric settings due to religious and social values, and a lack of rigorous and structured training for residents in providing person-centered care to SM. Consultant psychiatrists and residents attributed homosexuality to either Western values or classified it with pedophilia, or a cognitive distortion amenable to cognitive therapies. There is a need to unlearn stigma and improve the understanding of psychiatrists through training for the provision of community-sensitive services to sexual minority men.

## Introduction

Sexual minorities have historically been criminalized across many countries of the world, condemned by religions, and faced social stigma. According to the Human Dignity Trust, sixty-five countries criminalize consensual same-sex behavior between men. At the same time, twelve of these have the death penalty in their legal system for same-sex relationships [1]. These countries include Afghanistan, Brunei, Iran, Saudi Arabia, Pakistan, Yemen, United Arab Emirates, Qatar, Mauritania, Nigeria, Somalia, and Uganda. All of these countries, except Uganda, have a Muslim majority [1]. The stress of a lack of opportunities for companionship and a lack of a sense of community makes sexual and gender minority men vulnerable to common mental illnesses [2]. Based on a national survey from twelve countries, gay and bisexual men were twice as likely as heterosexual men to report a mental health condition [3]. A recently conducted meta-analysis has shown that the prevalence of depression among men who have sex with men (MSM) is 35% with the highest prevalence in Asia (37%) [4]. It should be noted that this is manifold above the global prevalence of depression, which is 4% [5]. The psychiatric diagnostic systems have pathologized and discriminated against people with a homosexual orientation until the 1990s [6]. Various conversion therapies were claimed to ‘cure’ people of ‘homosexual orientation’ [7]. Psychiatrists may share stigmatizing values with society; thus, sexual minority men (SM) have unsatisfying experiences with mental health professionals, including psychiatrists [8,9].

Pakistan is also one of the twelve countries where the death penalty for homosexual relationships is possible. Pakistan also ranks 102nd among 175 countries in the Global Acceptability Index (GAI) for Lesbian, Gay, Bisexual, and Transgender (LGBT) people. GAI is a composite index reflecting public beliefs and policies related to the LGBT community in a country. From 1980-2020, the GAI of Pakistan has remained the same, and there have been no major changes in acceptability or policy related to sexual minority men, though transgender people have most recently been recognized legally [10,11]. There is a dearth of research on social stigma and discrimination faced by sexual minority men in Pakistan. The most recent and the only study reporting stigma at the national level is the Integrated Biological and Behavioral Surveillance 2016–2017 [12]. According to the study, 31.3% of MSM face discrimination, 51.9% are physically hurt, and 18.5% are incarcerated. In addition, 6.3% of MSM report being denied healthcare services or treated unfairly [12]. There is only one study that reports depressive symptoms among MSM in Pakistan. The study had a small sample size and included MSM and transgender individuals employed in HIV organizations. According to the study, 9.89% had moderately severe and 7.6% had severe depressive symptoms (both MSM and TG) [13]. Though a higher prevalence of mental health conditions would suggest a higher need for mental health services, the barriers to such services are unassessed [13]. Power dynamics exist between medical professionals and patients. Traditionally, medical professionals have enjoyed power by being representative of a patriarchal culture, wealthy status, and informational power, which patients may lack. These skewed power dynamics play their role in interpersonal interactions between a medical professional and the patient. When the values of the patient and the medical professional contradict each other, the medical professional may take a paternalistic attitude, including negating patients’ concerns, and in the case of sexual minority men, dismissing their identity [14]. Rees et al, have conducted an extensive review of qualitative studies to identify LGBT service users’ experience of mental health services. They identified stigma and a lack of understanding of LGBT issues among mental health practitioners. The service users reported use of cis-heteronormative language and an expectation to appear cis-normative [15]. The users also reported homophobia and heterosexist bias expressed by mental health professionals. Such behaviors are detrimental to the relationship between the service user and medical professionals [15]. To overcome these barriers to providing client-centered services, various professional bodies have recommended training for medical professionals, including the American Medical Association, the American Nursing Association, and the American Psychological Association [16,17]. However, there are no such training recommendations in Pakistan, including for psychiatrists [18]. This lack of training and social stigma adds to health barriers to accessing mental health services [19,20] The objectives of the current study are to identify the capacity needs of the psychiatry fraternity for providing person-centered and non-stigmatizing psychiatric care to sexual minority men in Pakistan.

## Materials and methods

A qualitative exploratory study was conducted from May 2024 to October 2024.

### Ethics statement

The protocol of the research was approved by the Ethics Review Committee of Sheikh Khalifa bin Zayed Al Nahyan Hospital, Pakistan (No. 121/SKBZ/ CMH/Rkt); Dated: 04 April 2024.

### Participants

We recruited participants in three groups. This included consultant psychiatrists, postgraduate residents in psychiatry, and sexual minority men. Consultant psychiatrists who were exclusively trained in Pakistan and approved supervisors for a postgraduate residency program were interviewed. Postgraduate psychiatry residents (PGRs) currently teaching in Pakistan were recruited. Any PGR who has previously trained outside of Pakistan was excluded. The reason for exclusion was to include the perspective of locally trained psychiatrists. Self-identified sexual minority men, eighteen years of age or older, who had experience in availing psychiatry services from Pakistan were included. SM, who was under the clinical care of the authors, was excluded from the study, as this may influence reporting among the study participants. Written consent was taken from all participants.

### Participant recruitment

Consultant psychiatrists through invitations sent from the principal investigator AU. A total of five consultant psychiatrists were approached of which four agreed for inclusion. For PGRs we sent invitation flyer in the national resident’s group on the social media. A total of nine PGRs volunteered and all were included after eligibility screen. SM were recruited from the health organizations working specifically for the health of gay and sexual minority men in Pakistan (primarily on HIV prevention). An invitation to participate in the study was displayed in the organizational office and social media of the community organizations. Only individuals volunteering to interview contacted the PI, who performed an eligibility screen. Around ten people volunteered of which eight met the eligibility criteria and were included. Data was analyzed simultaneously after each round of interviews (generally 6–8 interviews). Recruitment was stopped after reaching saturation when no new themes emerged. A total of three rounds of interviews were conducted. Analysis was conducted as mentioned below.

### Data collection instruments

Semi-structured in-depth interviews were conducted based on an interview guide developed separately for three groups. The interview guide was developed based on the existing literature by the PI (AU), followed by elaboration between the research team. The draft of the interview guide was shared with the staff of the SM organization. Written feedback was taken for all three interview guides by the SM organization and incorporated into the revised version. Interviews for all groups were conducted in the Urdu language and recorded on a recording device. To explore the capacity needs of the psychiatry fraternity, psychiatry consultants and PGRs were asked about their understanding of common mental health needs of SM, experience dealing with patients with SM, conflict between personal values and professional ethics, and perceived capacity needs of the psychiatry fraternity. Clinical scenarios were developed by the PI in collaboration with HMA. HMA is a professorial instructor in psychiatry and a medical educator. Three scenarios were included: one of an adult gay man who requested conversion therapy, the second for a gay man with depressive symptoms, and the third with a gay man who has internalized homophobia due to the personal religious values conflicting with sexual orientation. These scenarios were used to engage psychiatrists in discussion and explore their understanding of ethical standards of service delivery to SM and their position on conversion therapy. SM participants were asked about their experiences in psychiatry services as well as recommendations for providing community-centered services to them in the Pakistani context.

### Data analysis

Interviews were transcribed in Urdu by one author (MK), followed by manual translation (UA) of the transcript into the English language (TN). Transcripts were back-translated to Urdu by one author (BR) to check and rechecked independently by a second author to ensure linguistic accuracy and preservation of meaning AA. HAM supervised the whole process. Transcripts were coded using description and interpretation-focused coding by two independent researchers AU and MK as described by Philip Adu [20]. Description-focused coding is assigning empirical codes to data, mostly a noun to summarize text in the data. Description-focused coding is used to capture behavior and experiences. This method of coding allows researchers to bracket their personal experiences and minimize bias. We also employed interpretation-focused coding for coding the responses of psychiatrists to clinical scenarios. Interpretation-focused coding is informed by the hermeneutic approach. Both coders individually coded the data; any differences were mutually discussed and agreed upon. This was followed by the grouping of codes into subthemes and themes. The grouping of codes into themes was guided by a presumption-focused categorization strategy, where each subtheme or theme represents a presumption made by the researcher, followed by empirical evidence from the code book and transcripts. Themes repeated frequently or that have theoretical importance were retained [20]. Data management was done using NVIVO version 14.0.

### Reflexivity statement

AU and MK coded the data. AU (the principal investigator) has worked in psychiatry for three years and has worked in HIV public health response for six years. This included working as a manager for a community organization working on the rights of the SM community. AU has advocated for an increased role of the SM community in the HIV public health response, as well as advocated for access to mental health services for SM. MK has worked in psychiatry for two years. Reflexivity notes were shared and reviewed by HAM, supervising the project. HAM is trained in the US as a psychiatrist and has undergone rigorous cultural competence training related to SM. AH has been working in Pakistan for the last 15 years.

## Results

We interviewed 4 consultant psychiatrists (CP), 9 postgraduate residents (PGR) in psychiatry, and 8 sexual minority men (SM). The sociodemographic characteristics of participants are summarized in Table 1. It should be noted that among the 8 SM included in the study, 4 SM were medical doctors by profession.

**Table 1:** Sociodemographic characteristics of sexual minority men, postgraduate residents in psychiatry and consultant psychiatrists (N = 21), Pakistan 2024.

Sociodemographic characteristic	Consultant psychiatrist (n = 4)	Postgraduate resident in psychiatry (n = 9)	Sexual and minority men (n = 8)
Mean age in years (range)	44.5 years (39–53)	29.5 years (27–33)	34.1 years (20–49)
Sex at birth			
Male	2	4	8
Female	2	4	0
Religion at birth			
Islam	4	8	7
Christianity	0	0	1
Mean years of experience in psychiatry (range)	17.5 years (16–20)	3.1 years (3 months-6 years)	–
Marital status (heterosexual marriage)			
Married	4	6	1
Not married	0	2	7
Homosexual partner status			
Partnered	–	–	3
Not partnered	–	–	5
Aware of Section 377, Pakistan Penal Code	0	0	–
Education			
High school	–	–	1
Graduate	–	–	1
Post-graduate	–	–	6
Income in PKR	–	–	185,000

Based on thematic analysis, four recurrent themes and ten sub-themes have emerged. Themes, subthemes, codes, and participants contributing are given in Table 2.

**Table 2:** Thematic analysis and summary codes based on qualitative interviews of sexual minority men, postgraduate residents in psychiatry, and consultant psychiatrists (N = 21), Pakistan 2024.

Themes	Subthemes	Codes	Participants
Lack of understanding among psychiatrists related to the human rights of SM, and confidentiality	Breach of the personal space of SM by the psychiatrists	Psychiatrists can be intrusive in history-taking	SM 4, SM 6
	Lack of understanding of human rights	Lack of understanding about confidentiality breach in case of STIs	CP 1
		Lack of understanding of the right to love	CP 1
Lack of knowledge about sexuality and gender	Psychiatrists’ lack of theoretical knowledge about sexuality, sexual orientation, and gender identity	Psychiatrists’ lack of understanding of the difference between sexual orientation and gender identity	CP 2, CP 3, PGR 1, PGR 4, PGR 6, PGR 7, PGR 9
		Psychiatrists understand gender but not sexuality	PGR 6
		Psychiatrists lack understanding of sexual orientation	PGR 9, SM 3
		Psychiatrists’ lack of understanding of ego-dystonic homosexuality	PGR 1, PGR 6, PGR 8, PGR 9
		Psychiatrists attribute homosexuality to a cause such as lack of exposure to women, childhood abuse, compulsive behavior, etc.	CP 2, CP 3, PGR 8
		Psychiatrists confuse homosexuality with pedophilia	CP 2, PGR 3, PGR 6
	Lack of theoretical knowledge on guidelines and the position of ethical psychiatry among psychiatrists	Lack of clarity on whether to recommend conversion therapy or not	PGR 7
		Conflicting ideas about whether to validate homosexuality or not	PGR 4
		Lack of theoretical knowledge on what sexual orientation is	PGR 9
		Lack of theoretical knowledge on guidelines and the position of ethical psychiatry	PGR 7
	Psychiatrists aimed to treat homosexuality	Psychiatrists recommended conversion therapy	CP 2, CP 3, CP 4, PGR 2, SM 4
		Psychiatrists recommended abstaining from homosexual sex but not sexual feelings, as a midway solution	PGR 3
Stigma in psychiatric settings due to religious and social values		Homosexuality conflicts with my religious values as a clinician	CP 4, PGR 2, PGR 3, PGR 9
		Perceived stigma due to the religious values of psychiatrists among SM	SM 1, SM 2, SM 8
		My religion invalidates homosexuality	PGR 4, PGR5
		Social stigma among psychiatrists against SM	CP 2, CP 3, CP 4, SM 3, PGR 5, PGR4, PGR7
		Lack of respect among PGRs for SM patients (cracking jokes etc.)	PGR7
Medical education and training gaps	Social stigma deters discussion by supervisors with PGRs	Supervisors feel threatened by not discussing these topics in detail due to stigma	CP 2, CP 3
		The mental health needs of SM are not discussed between PGRs and supervisors due to stigma	PGR1
	Lack of capacity in supervisors to offer support to PGRs	Lack of capacity of supervisors to deal with such cases	PGR 2, PGR 5
	Unstructured postgraduate training, lack of support and discussion on clinical management, and unlearning stigma among PGRs against SM	Lack of support by supervisors to PGRs in dealing with SM	PGR 2, PGR 5
		Lack of discussion between PGRs and supervisors on sexuality	PGR 4, PGR 8
		Lack of training of PGRs in the clinical management of mental health conditions related to SM	PGR 5, PGR 9
		Lack of training of PGRs to separate conflicting socio-cultural and religious values from psychiatry	PGR 2, PGR 3, PGR 5, PGR 6, PGR 7, PGR 8
		Postgraduate training in psychiatry is unstructured	CP 3
	Lack of management skills in dealing with mental health conditions, empathizing, counseling, and overcoming hesitation while dealing with SM.	Lack of skills to take history, and advising treatment	PGR 2, PGR 5, PGR 7, SM 7
		Lack of skills to empathize or relate to SM	PGR 3, PGR 4
		Lack of counseling skills	PGR 4
		Hesitation dealing with SM	PGR 4

### Thematic area 1. Lack of understanding among psychiatrists related to the rights of privacy and confidentiality

The SM interviewed stated that the treating psychiatrists were unaware of their human rights. The theme also emerged during an interview with a consultant psychiatrist. SM found the history taking on sexuality by the psychiatrist as intrusive and crossing boundaries. An excerpt from an SM interview is given below.

*He went through a series of questions which were not about sexual identity but my activity, like what I have done or not. I was curious about why they were important, but I still went through those. SM 4, 48 years 48-year-old*

A consultant psychiatrist also mentioned breaching confidentiality and disclosing sexual relationships to the wife based on the perceived risk of HIV.

*Whether it’s a homosexual or an intravenous drug abuser, guidelines are very clear. One must confront the patient about barrier methods; if the patient agrees and follows, then that is acceptable; otherwise, confidentiality may be breached. CP 1, 43 years old, male consultant with 12 years of experience as a consultant*

### Thematic area 2. Lack of knowledge about sexuality and gender

A major theme that emerged during data analysis was the lack of understanding about sexuality and gender. Participants, psychiatry residents, or psychiatrists interviewed were not clear on terms such as gender identity, sexual behavior, or sexual orientation when probed during the interview. A year four resident, when asked about definitions of sexual orientation and gender identity, could not provide a clear response.

*Sexual orientation refers to whether we are male or female, I think. And gender identity, that is, our gender identity, means the gender that is assigned to us. I think that’s what it is. I’m not sure if I’m confusing them. PGR 7, 29-year-old female, resident year 4*

Another resident who has completed membership of the psychiatry college in Pakistan, and in the last year of residency of fellowship training, confused homosexuality with pedophilia.

*Yes, exactly. Whether they are homosexual or heterosexual. Or when you go into subtypes, like pedophilia. All these things fall under the types of sexual orientation. PGR 6, 31-year-old female, resident year 6*

The psychiatrist also thought of homosexuality as a disease. They would attribute homosexuality to ‘cognitive distortions’ and thus amenable to cognitive therapies. The theme was recurrent, and even consultant psychiatrists had similar opinions. A head of department and associate professor of psychiatry considers homosexuality treatable by psychotherapy.

*If they want to change, then it is a very good change; we are there to help them. CP 2, 39-year-old female, with 10 years of experience as a consultant*

Another head of department and associate professor of psychiatry also believed that ‘normalcy’ can be achieved using disputing techniques in psychotherapy.

*Suppose someone wants to revert to normalcy, as we’ve seen with some gender identity cases. In that case, there are instances where individuals who don’t feel attraction to the opposite sex, but due to religious or family pressure, they want to change their thoughts. This can be challenging. But we can dispute their thoughts just like we do in psychotherapy for depression. CP 3. 53 years old, female with 13 years of experience as a consultant*

One SM who had struggled with his sexual orientation turned to a psychiatrist for help and requested conversion therapy. The treating psychiatrist had provided so-called therapeutic interventions as conversion therapy. As a result of the traumatizing conversion experience, the person became skeptical of psychiatric care and would not seek help later, even when in need. It is worth noting that this man was also a medical doctor, thus showcasing that even as a medical doctor, SM had been subjected to non-evidence-based practices.

*I told them that I am gay and if this can be changed. That doctor immediately told me yes, we can do this (change sexuality), which made me think that I have more than one mental disorder. He went through a series of interventions such as hormone therapy, counselling, and what he called psychotherapy. SM 4, 48-year-old*

Another SM who has not gone through conversion therapy has also shared his experience. He has mentioned heteronormativity in Pakistani culture, a complete lack of awareness, and a lack of conversation on gender and sexuality. According to him, the psychiatrist shares the communal notion that homosexuality is something only existing in the West, or that people turn homosexual because they cannot have a relationship with women due to social and religious sanctions in Pakistan for heterosexual relationships.

*Psychiatrists, especially in Pakistan, often lack knowledge. In contrast, in Europe and the West, even though straight people still exist, there is more awareness about homosexuality and gay individuals. Here, the conversation doesn’t happen at all, and straight people might think that homosexuality is something only found in the West. Even if it’s present in Pakistan, they might assume that someone is abusing because they can’t find a girl, and “he is not a proper gay.” SM 3, 39 years old*

### Thematic area 3. Stigma in psychiatric settings due to religious and social values

Several of the SM interviewed, CP, and PGRs have stated that stigma is a key barrier to the provision of services. At times, it was noted by the CP and PGRs themselves in terms of ‘hesitation’ or ‘lack of comfort’. The origins of stigma are religion, as it has emerged recurrently in interviews with participants; one participant even thought that validating homosexual feelings may make her responsible for taking their patients to hell. It should be noted that all of the participant psychiatrists, either consultants or residents, were Muslim by religion.

*Certainly, it’s like this. A thought that stops me is whether I tell them that it’s okay to pursue this feeling (homosexuality), whether I will be taking them to hell? or something like that. PGR 4, 27-year-old female, resident year 2*

Another resident who has completed 2 years of training has explained the origins of his stigma from Quranic texts and referred to the incident of Lot, where a whole town faced the wrath of God due to homosexuality. He went a step further from sin and called homosexuality a crime, punishable in Islam

*I, as a Muslim and a practicing Muslim, was taught that in the Quran, homosexuality is absolutely a sin. It is a crime, these are the words that came to my mind after listening to homosexuality…Qur’an does not allow this thing, this thing has been negated, and this thing is such that the whole nation –Qom e Loot was punished. PGR 9, 30-year-old male, resident year 3*

Another resident of year 1 stated that even though sexuality cannot be changed, it is *haram* or forbidden, and that is the essence of worldly challenges that God makes us go through. According to her, homosexual acts or sex can be *controlled,* though not *changed*.

*It cannot be changed, but yes, we can try to control it. Even though we have no control over our thoughts, we can still exert control over our actions, and we are answerable for those actions. PGR 3, 27-year-old female, resident, year 1*

The stigma and religious values permeated the clinical interactions. A SM has shared an experience of being prescribed Quranic verses as part of their treatment for homosexuality. Since he had different religious affiliations, and he believed that religion invalidates his feeling of being gay, he saw this as stigmatizing and not under how ethical psychiatry is practiced globally.

*…. I would say, psychiatrists are very judgmental, ….. I have seen two or three psychiatrists in mind whose names … They do not see that disease as disease and non-disease as non-disease, but they try to evaluate by “Read this Surah (Chapter), recite this Darood, and then you’ll get better- SM 3, 39 years old*

### Thematic area 4. Medical education and training gaps

Psychiatry consultants and PGRs both mentioned the training needs and gaps in medical education for sensitizing psychiatrists on mental health needs and community sensitivity of sexual minority men. However, due to stigma, the conversations were avoided by consultants as well as PGRs.

*I’m telling you, we don’t discuss these topics in too much detail (because of religious stigma), but we do tell them (PGRs) that, according to the book, this is how it is, so work on it and observe where the patient’s thoughts are going, and to what extent we can help them. CP3, 53-year-old female with 13 years of experience as a consultant*

The psychiatrists also feared public backlash if they started capacity-building or training workshops on improving capacity and overcoming training gaps in the mental health of SM.

*If I see the environment of my hospital, yes, it is hostile; we have to be cautious about a lot of things. There is a difference; training and workshops can be done elsewhere, but here, due to religion, and threats from religious pressure groups who can go on strikes, we do not do anything visible. CP 2, 39-year-old female, with 10 years of experience as a consultant*

However, in addition to a lack of conversation, there was also a lack of capacity among consultants to provide support to budding psychiatrists. The excerpt below shows PGRs’ evaluation of the capacity of consultants. We also noted a lack of capacity among consultant psychiatrists, as evident in the themes mentioned above.

*She (my supervisor) mentioned that neither the patient was comfortable sharing this information, nor was she well-versed in how to deal with this patient or what questions to ask. PGR 2, 28-year-old male, resident year 2*

In addition to the lack of capacity, there is a lack of structural learning in training. Any discussion on homosexuality between residents and consultants was on a case-by-case basis. It was also highlighted by the consultant psychiatrist that there is a lack of a rigorous curriculum that has to be followed for the training of PGRs. In the absence of a rigorous curriculum, the training is very variable, and some trainees may not have adequate exposure or discussion to such cases. A trainee in the second year of her training stated no opportunities when SM mental health was discussed in the ward. She recalls one experience in two years of training.

*No, never. I would say there was a case conference one time in which there was a case about hepatitis being transferred by oro-anal contact in homosexual men. That was the only time we discussed homosexuality in the ward. PGR 4, 27-year-old female, resident year 2*

The problem of a lack of structured training curricula is not specific to issues around sexual minority men. The training program as a whole is not structured, as it is shared by a consultant supervisor.

*The advice we provide to our PGRs is not structured, like following a curriculum. CP3, 53-year-old female with 13 years of experience as a consultant*

There was a lack of knowledge of current practice recommendations on dealing with homosexuality. A female PGR who has almost completed her psychiatry residency stated her lack of understanding, as evident from her statement below.

*Well, if I talk about myself, I completed my psychiatry training in an institute for four years. We discussed some cases, but at the end of the day, like during this conversation, I understand that I didn’t know the proper guidelines. So, we should at least receive a (formal) training so that if someone asks me, I will have an answer. PGR 7 29 years female, resident year 4*

A SM who had experience in psychiatric care shared his experience in a psychiatric setting. He highlights that when the psychiatrist was unable to provide expected care, they started making homophobic remarks. This showcases that the training gap, lack of understanding, and stigma go hand in hand, with the former leading to the latter.

*Obviously, they try to get to the root cause, so they take you to a triggering position where all your emotions are triggered, then they slowly calm you down…. She failed to resolve it (when my anxiety was triggered) ….She couldn’t explain that and ended up making a homophobic remark. SM 7, 31 years old*

## Discussion

Our study highlights the capacity gaps among the psychiatry fraternity in providing services to sexual minority men. The key themes highlighted show that there is a lack of understanding and theoretical knowledge on the subject. Morris et al. have explored the barriers to mental health services among sexual minorities in the UK. The authors identified three major themes of barriers, which included practitioner-centered barriers to access to services [19]. The study participants reported a lack of understanding of the stress of sexual minorities, and community dynamics were key barriers to accessing mental health care. However, the nature of the lack of knowledge of psychiatrists in our study is different from that reported by Morris [19]. In our study, the psychiatry fraternity lacks awareness about gender identity, sexual orientation, or sexual behaviors, whereas the results from Morris cite a lack of ‘awareness’ of the nature of stressors people from a sexual minority face.

The psychiatry fraternity in Pakistan also holds a pathologized model of homosexuality, viewing it as a mental disorder or sin. Stigma has also emerged among the psychiatry fraternity as a limiting barrier to the extension of humanistic services. Religion is a key driver of stigma against sexual and gender minorities. Chapman et al. have explored factors associated with attitude, knowledge, and beliefs among medical students and nursing staff [21]. In this study, ‘religiosity’ was associated with lower knowledge about lesbian, gay, bisexual, and transgender (LGBT) people. Fischer et al. conducted a cross-sectional study among healthcare workers and the general population. The authors of the study reported higher homophobia and transphobia among those who were religious [22]. Page et al. have compared heteronormativity among people from eight different religions residing in the UK and Canada. Among believers of all religions, Muslims were the highest in terms of believing in heteronormativity. Muslims were also the least likely of all believers of all religions to believe in equality between homosexuals and heterosexuals [23]. Geurts et al., however, argue that it is not religiosity but the belief that they are keepers of paradise, i.e., willingness to judge, that fuels homophobia among Muslims [24]. Another theme in our study was the lack of awareness of psychiatrists about the human rights of sexual minority men. Under this theme, participants reported being asked intrusive questions by the psychiatrists. Eday et al. have reported that bisexual men and women faced similar issues with mental health professionals in Canada [16].

In our study, a key barrier to the provision of humanistic services to sexual minority men was the lack of structured training. Psychiatry as a field is fledgling in Pakistan, with only 400 psychiatrists in a country of 220 million people. Postgraduate training is largely regulated by the College of Physicians and Surgeons, Pakistan [25]. The curriculum of training in psychiatry by the College of Physicians and Surgeons does not mention training on gender and sexuality during the four years of training [26,27]. Ho et al., have evaluated the provision of training on sexual orientation to clinicians in NHS talking therapies. The authors recommend implementing minimum contact hours of training on the subject matter and discussion of theoretical aspects in more detail, with an appreciation of diversity within sexual minorities [28]. Morris et al., have published a systematic review of training interventions to reduce bias against LGBT individuals among medical students and professionals. Key interventions focused on improving the knowledge and comfort level of medical professionals or decreasing implicit or explicit bias. Methods of training delivery included lectures, conferences, workshops, group discussions, and problem-based learning. The training included delivery by not only health professionals but also by LGBT community speakers sharing their experiences or delivering a presentation [29]. Sekoni et al., conducted a systematic review to explore the effect of training on LGBT issues for medical students and professionals [30]. Most of the training initiatives were quasi-experimental in nature. There was consistent improvement in knowledge, increased comfort, and acceptance among medical professionals after training [30].

A major limitation of the study was that half of the study participants, sexual minority men, were doctors by profession. Since these participants were medical professionals, they may face stigma in psychiatry services, which is different from that faced by other sexual minority men. In addition, the monthly income and education of the SM men included were higher than average Pakistani men, which may interact with stigma and reported themes. We also interviewed only four consultant psychiatrists, who were all based in one city, which may limit the generalization of the findings. In addition, only psychiatrists trained in Pakistan were included in the study while those who have received elsewhere were excluded. Thus, the reflects capacity needs of the locally trained psychiatrists. In our study, the PGRs recruited were from all provinces and included both males and females. Our study is also the first from the country to explore the topic.

Our study highlights that there is a lack of capacity among consultant psychiatrists and PGRs to provide community-sensitive services to sexual minority men. There is also a lack of a prescriptive curriculum and training structure. Added to this is a highly prevalent religious stigma among psychiatrists. It is recommended for developing curricula on theoretical aspects, clinical skills, and human rights, as well as de-stigmatizing sexual minorities in the psychiatry fraternity, to be able to provide community-sensitive services.

## Supporting information

S1 File Codebook. (XLSX)

S1 Data Data. (XLSX)
